# Cinnamaldehyde induces autophagy-mediated cell death through ER stress and epigenetic modification in gastric cancer cells

**DOI:** 10.1038/s41401-021-00672-x

**Published:** 2021-05-12

**Authors:** Tae Woo Kim

**Affiliations:** Department of Preventive Medicine, College of Korean Medicine, Kyung Hee University, Seoul, Republic of Korea

**Keywords:** cinnamaldehyde, ER stress, autophagy, cell death, G9a

## Abstract

Previous reports suggested that cinnamaldehyde (CA), the bioactive ingredient in *Cinnamomum cassia*, can suppress tumor growth, migratory, and invasive abilities. However, the role and molecular mechanisms of CA in GC are not completely understood. In the present study, we found that CA-induced ER stress and cell death via the PERK–CHOP axis and Ca^2+^ release in GC cells. Inhibition of ER stress using specific–siRNA blocked CA-induced cell death. Interestingly, CA treatment resulted in autophagic cell death by inducing Beclin-1, ATG5, and LC3B expression and by inhibiting p62 expression whereas autophagy inhibition suppressed CA-induced cell death. We showed that CA induces the inhibition of G9a and the activation of LC3B. Moreover, CA inhibited G9a binding on Beclin-1 and LC3B promoter. Overall, these results suggested that CA regulates the PERK–CHOP signaling, and G9a inhibition activates autophagic cell death via ER stress in GC cells.

## Introduction

*Cinnamomum cassia* (called Chinese cassia or Chinese cinnamon) is an evergreen tree that is widely found in southern and eastern Asia [1]. Cinnamaldehyde (CA) is one of the most important bioactive ingredients in *C*. cassia and is often used as traditional Chinese medicine [2]. CA has diverse biological functions including anti-oxidant, anti-inflammation, anti-diabetic, and anti-cancer effects [3–6]. CA is a natural flavonoid, and flavonoids like luteonin, quercetin, and CA are powerful natural anti-cancer compounds that function via the inhibition of histone deacetylase (HDAC) [7–9]. Emerging HDAC inhibitors that induce powerful apoptosis and cell death in various cancer cell types have emerged as potent anticancer agents that can restore programmed cell death in malignant cells [10]. HDACs are upregulated in many cancer cells, and HDAC inhibition regulates cell death or growth-related genes via acetylation and methylation of histones [11]. HDAC8 has been studied to play a potential role for tumor growth and proliferation, whereas HDAC8 inhibition caused apoptosis, cell death, and cell cycle arrest in various cancer types [12].

Histone methylations via epigenetic modification on lysine 4, 9, and 27 of H3 play a potential role as cell survival or death codes and perform transcriptional activator and inhibitor activities for histone-bound genes [13]. HDAC inhibitors can frequently suppress histone methyl transferases, such as G9a and EZH2, and their inhibition regulates methylation on lysine 9 and 27 of H3 [14]. G9a is reportedly involved in cell development, cell differentiation, cell cycle, and autophagic cell death [15, 16]. G9a normally binds on LC3B promoter, but repressive binding complex with RFX5, Sin3B, and HDACs binds on LC3B promoter and blocks LC3B expression [17, 18]. Targeting HDAC regulates autophagy-related genes by inhibiting G9a [19]. BIX01294, selective inhibitor of G9a/EHMT2, could powerfully mediate autophagy and apoptosis through the mTOR/AMPK/ULK1 pathway in various cancer types, such as gastric, breast, oral squamous carcinoma, glioma, and neuroblastoma [20–22]. G9a inhibition may be a potential anti-cancer therapeutic strategy and a promising tumor therapeutic target.

Endoplasmic reticulum (ER) has potential function in protein translocation, folding, and maturation [23]. Disruption of ER function by diverse stimuli and pathological conditions induces ER stress by increasing misfolded or unfolded proteins [24]. Emerging reports suggested that severe or excessive ER stress causes cell death in various diseases via powerful, anticancer-related molecular mechanisms [25]. In particular, sustained intracellular calcium (Ca^2+^) release in ER lumen resulted in ER stress-induced cell death via Ca^2+^ interacting chaperones such as GRP78/Bip [26]. ER stress regulates diverse signaling pathways via the activation of three ubiquitous branches; PRKR-like ER kinase (PERK), inositol-requiring enzyme1 alpha (IRE1α), and activating transcription factor 6 (ATF6) [27]. In normal conditions, GRP78/Bip binds to unfolded protein response (UPR) such as PERK and IRE1α and inhibits ER stress signaling. However, under ER stress, GRP78 dissociates with UPR sensors [28]. With PERK release by ER stress, PERK phosphorylates eukaryotic translation initiation factor-2α (eIF2α), and its phosphorylation stimulates the activation of activating transcription factor-4 (ATF4) in the cytosol and –CCAAT-/enhancer-binding protein homologous protein (CHOP) in the nucleus [29]. Activated IRE1 regulates the splicing of the stress response transcription factor X-box binding protein-1 (XBP-1), binds to c-Jun*-N-*terminal inhibitory kinase (JNK) and recruits CHOP and TRAF2, which modulates the induction of ER stress-mediated cell death mechanism [30]. Recent reports on the relationship between ER stress and autophagy suggested autophagic cell death as the underlying mechanism of ER stress-induced cell death [31]. During ER stress, activated PERK mediates the phosphorylation of eIF2α and activation of ATF4, and cytosol ATF4 binds the LC3B promoter and activates autophagic cell death-related mechanism by upregulating LC3B, an autophagy-related gene [32, 33]. Furthermore, various flavonoids were recently reported to have anti-cancer effects via ER stress and autophagy [34]. However, the mechanisms by which CA induces ER stress and autophagic cell death in cancer are not well understood.

Autophagy plays a potential role in the self-cleaning process involving degradation and recycling of cellular components [35]. In cancer, autophagy has paradoxical roles, such as tumor inhibition and tumor survival [36]. Several studies have proposed that PERK-mediated ATF4 activation regulates autophagy-related genes, including Beclin-1, LC3-II, and p62, and ATF4 inhibition downregulates Beclin-1 and LC3-II and upregulates p62 [37]. Both Bcl2 and Beclin-1 are central regulators in autophagic cell death, and Bcl-2 overexpression inhibits Beclin-1-related autophagy [38]. Previous reports have studied that ER stress-induced eIF2α/ATF4 axis induces AMP-activated protein kinase (AMPK) phosphorylation leading to autophagy and cell death [39]. Furthermore, PERK–eIF2α–CHOP-dependent signaling pathway leads to transcriptional induction of LC3B and ATG5 for autophagosome formation of the autophagy process [40]. However, the mechanisms by which CA induces cell death are not well understood.

In the present study, we sought to test whether CA induces autophagic cell death via ER stress and histone change in GC. We identified that CA mediates autophagic cell death via the PERK–eIF2α–ATF4–CHOP axis and HDAC/G9a pathway in GC, thus broadening our understanding of CA as a novel anti-tumor compound.

## Materials and methods

### Reagents

Cinnamaldehyde (CA; W228613), cisplatin (1134357), paclitaxel (T7191), (Z-VAD-FMK; caspase inhibitor; V116), 3-methyladenine (3-MA; autophagy inhibitor; M9281), chloroquine (CQ; autophagy inhibitor; C6628), compound C (AMPK inhibitor; P5499), SBI-0206965 (ULK1 inhibitor; SML1540), BIX-01294 (G9a inhibitor; B9311), and thapsigargin (TG; ER stress inducer; T9033) were purchased from Sigma-Aldrich (St. Louis, MO USA).

### Cell culture

The human GC cell lines (SNU-638, SNU-216, AGS, NCI-N87, MKN-45, and MKN-74) were purchased from the Korean Cell Line Bank (Cancer Research Center, Seoul National University, Seoul, Korea). Cells were cultured in RPMI-1640 medium (Welgene) supplemented with 10% fetal bovine serum (JR Scientific) and 100 μg/mL antibiotics (100 U/ mL penicillin and 100 μg/ mL streptomycin, Welgene) in a 5% CO_2_ humidified incubator at 37 °C.

### Cell viability assay

The WST-1 assay was performed according to the manufacturer’s instructions (Roche, Mannheim, Germany; 11644807001) with 10 μL of WST-1 reagent added to each well of a 96-well plate (1 × 10^4^ cell/well). To determine the cell viability in the supernatant, the conversion of WST-1 reagent into chromogenic formazan was measured and analyzed with a microplate reader (Molecular devices, USA).

### LDH assay

Cells (1 × 10^4^ cells/well) were seeded into a 96-well plate with RPMI-1640 growth medium. To determine the LDH (Thermo Scientific Pierce; 13464269) activity in supernatants, 100 μL of the reaction mixture was added and incubated for 30 min in a dark room at room temperature. The LDH activity was measured by the absorbance of the samples at 490 or 492 nm using a microplate reader (Molecular Devices, USA).

### Transfection

NCI-N87 and MKN-74 cells (3 × 10^5^ cell/well) were transfected with double-stranded siRNAs (30 nmol/mL) of LC3B (Santacruz; sc-43390), ATG5 (Santacruz; sc-41445), PERK (Santacruz; sc-36213), G9a (Santacruz; sc-43777), and CHOP (Bioneer; 1649-1) in a six-well plate for 24 h by the Lipofectamine 2000 (Invitrogen; 11668019) method according to the manufacturer’s protocol and were then recovered in RPMI-1640 medium (Welgene) containing 5% fetal bovine serum (Gibco) and 100 μg/mL antibiotics (100 U/mL penicillin and 100 μg/mL streptomycin, Gibco) for 24 h. After recovering, viable cells were measured and analyzed using the WST-1 assay.

### Isolation of protein

Protein cell lysates from cells (2 × 10^6^ cell/well) in a 100-mm cell culture dish were collected in RIPA buffer containing a protease inhibitor cocktail (Sigma; P8340) on ice for 30 min. And then Protein cell lysates were passed through an 18-gauge needle and spun down. The supernatant was analyzed for protein content using the BCA method (Thermo scientific, Pierce BCA Protein Assay Kit, USA; 23225).

### Western analysis

For Western blotting analyses, cells were solubilized in radioimmunoprecipitation assay (RIPA) lysis buffer [50-mM Tris-HCl (pH 7.4), 150-mM NaCl, 1% NP40, 0.25% sodium deoxycholate, 1-mM phenylmethylsulfonylfluoride (PMSF), 1-mM sodium orthovanadate, and 1× Sigma protease inhibitor cocktail], and protein content was measured using a standard bicinchoninic acid assay. Equal amounts of protein (20 μg) were size-fractionated by 8%–15% SDS-PAGE and then transferred onto a PVDF membrane (Millipore Corporation, Billerica, MA, USA; IPVH00010). Membranes were blocked by incubation for 30 min with 5% skim milk/PBS-T [PBS with 5% powdered milk (BD) and 1% Tween20 (Sigma; P9416)], and incubated overnight at 4 °C with primary antibodies diluted in 1× PBST buffer. The following primary antibodies were used: β-actin (sc-47778), Bcl-2 (sc-7382), Beclin-1 (sc-48341), ULK1 (sc-390904), GRP78 (sc-166490), eIF2α (sc-133132), and ATG5 (sc-133158) (Santa Cruz, 1:1000); LC3B (Sigma, 1:1000; L7543); p62 (Sigma, 1:1000; P0068); G9a (Abcam, 1:1000; ab40542); and cleaved caspase-3 (#9654), cleaved caspase-9 (#9505), p-AMPKα (Thr172; #50082), AMPKα (#2793), p-mTOR (Ser2448; #5536), mTOR (#2983), p-ULK1 (Ser555; #5869), PERK (#5683), p-PERK (Thr980; #3170), p-eIF2α (Ser51; #3398), and CHOP (#2895)(CellSignaling, 1:1000). The membranes were washed three times with PBST buffer. A secondary antibody diluted in PBST or TBST buffer was added, and incubation was done for 40 min at room temperature. The following secondary antibodies were used: anti-rabbit IgG HRP-linked antibody (KPL, 1:6000; 5450-0010) and anti-mouse IgG HRP-linked antibody (KPL, 1:6000; 5450-0011). The membranes were washed six times with PBST buffer for 1 h. The blots were visualized using Western chemiluminescent HRP substrate (Millipore; WBKLS0500).

### pEGFP-LC3 puncta

NCI-N87 and MKN-74 cells (2 × 10^5^ cells/well) in a six-well plate were transfected with pEGFP-LC3 using Lipofectamin 2000 (Invitrogen), and then treated with CA (50 μg/mL, 24 h) for 24 h. A pEGFP-LC3B puncta was observed by confocal microscopy. Confocal microscopy was performed using a ZEISS LSM5 PASCAL confocal microscope with 405 and 488-nm excitation lasers.

### Immunoprecipitation (IP) assay

We extracted cell lysates from NCI-N87 and MKN-74 cells (2 × 10^6^/well) on a 100-mm cell culture plate in an IP buffer (pH 7.5) containing 50-mM Tris-HCl, 250-mM NaCl, 5-mM EDTA, 0.5%(v/v) NP-40, and protease inhibitor cocktail (Sigma). We incubated anti-Bcl-2 (Santa Cruz) and anti-Beclin-1 (Santa Cruz) with lysate at 4 °C for 16 h. We used protein A/G plus agarose (Santa Cruz; sc-2003) to pull down immunocomplexes. We washed precipitates three times with IP buffer. We resolved the immunoprecipitated proteins with 12% SDS-PAGE and analyzed them.

### Chromatin immunoprecipitation (ChIP) assay

ChIP assays were performed using an EZ ChIP Chromatin Immunoprecipitation kit (Millipore, Billerica, MA, USA; 17371) as described in the supplier’s protocol. Briefly, the cross-linked chromatin was sonicated after cell lysis and then incubated overnight at 4 °C with antibodies against G9a (Abcam). The immunocomplex was precipitated with protein A–agarose (Millipore), and the beads were washed, sequentially treated with 10 µL of RNase A (37 °C for 30 min) and 75 µL of proteinase K (45 °C for 4 h) and then incubated at 65 °C overnight to reverse cross-link of the chromatin. The DNA was recovered by phenol–chloroform extraction and co-precipitation with glycogen and was then dissolved in 50 µL of Tris-EDTA (TE) buffer. DNA associated with the ER was amplified by PCR using 1 µL of precipitated DNA. PCR primers [5′-GAAGTGGCTATCGCCAGAGT-3′ (sense) and 5′-GCTGCTTGAAGGTCTTCTCC-3′ (antisense)] were designed to amplify the G9a binding site on the LC3B gene promoter and PCR primers [5′-CCCGTATCATACCATTCCTAG-3′ (sense) and 5′-GAAACTCGTGTCCAGTTTCAG-3′ (antisense)] were designed to amplify the G9a binding site on the Beclin-1 gene promoter. Quantitative PCR conditions were 40 cycles at 94 °C for 40 s, 60 °C for 1 min, and 72 °C for 40 s.

### Measurement of intracellular Ca^**2+**^ level

Calcium release assays were performed using a Calcium Assay Kit (Colorimetric) (Abcam; ab102505) as described in the supplier’s protocol. For the calcium release experiments, NCI-N87 and MKN-74 cells (1 × 10^4^ cells/well) were seeded and incubated into a 96-well plate with growth medium. Then, the cells were treated with CA. NCI-N87 and MKN-74 cells were washed with calcium-free buffer and then added to chromogenic reagent and calcium assay buffer for 10 min at room temperature. The fluorescence was measured and analyzed by the absorbance of the samples at 575 nm using a microplate reader (Molecular Devices, USA).

### Statistical Analysis

All results were confirmed in at least three independent experiments; Student’s *t*-tests were used for between-groups comparisons of the means of quantitative data, and *P* < 0.05 was considered statistically significant.

## Results

### CA suppresses the growth of GC cells

**Fig. 1:**
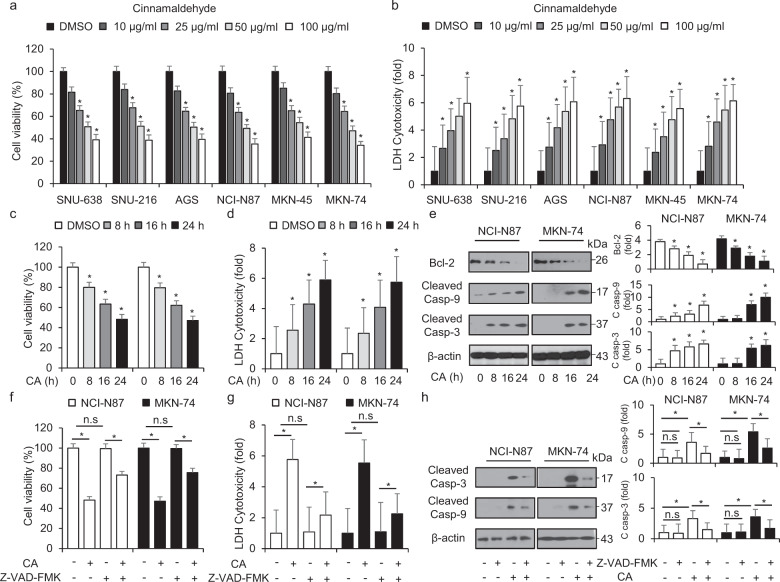
Anti-cancer effects of cinnamaldehyde in GC cells. **a**–**d** Cell viability and LDH cytotoxicity induced by cinnamaldehyde (CA) in GC cells, including SNU-638, SNU-216, AGS, NCI-N87, MKN-45, and MKN-74, measured using WST-1 and LDH assays in a dose-dependent manner (0, 25, 50, and 100 μg/mL; 24 h) and a time-dependent manner (0, 8, 16, and 24 h). Cell viability and LDH release of control cells were set at 100%; *P < 0.05. **e** Western blotting analyses of cleaved caspase-3 and -9 analyzed on the indicated times (0, 8, 16, and 24 h; 50 μg/mL) in CA-treated NCI-N87 and MKN-74 cells; *P < 0.05. **f**–**h** Effect of Z-VAD-FMK (50 μM) on CA-caused apoptotic cell death. NCI-N87 and MKN-74 cells were pretreated with Z-VAD-FMK for 4 h and were subsequently treated with CA (50 μg/mL, 24 h). Cell viability was determined using the WST-1 assay, and cell cytotoxicity was analyzed using the LDH assay; *P < 0.05. Total protein samples were analyzed by Western blotting analyses using apoptosis markers such as cleaved caspase-3 and -9. β-actin was used as a protein loading control.

To identify the cytotoxic effects of CA in GC, we performed the cell viability and LDH release using WST-1 assay and LDH assay at indicated doses. We found that CA triggers anti-proliferative effects through a dose-dependent decrease of cell viability and increase of LDH production compared with control in various GC cell types, including SNU-638, SNU-216, AGS, NCI-N87, MKN-45, and MKN-74 cells (Fig. 1a, b). In addition, it was found that the anti-proliferative effects of CA on NCI-N87 and MKN-74 cells were time-dependent and that CA mediates LDH cytotoxicity in GC cells (Fig. 1c, d). The cytotoxic effect was identified by the increase of caspase 3 and 9 cleavage and downregulation of Bcl-2 in NCI-N87 and MKN-74 cells (Fig. 1e). To evaluate whether CA-treated cytotoxicity was associated with apoptosis, we co-treated NCI-N87 and MKN-74 cells with CA and Z-VAD-FMK (50 µM), a pan-caspase inhibitor. Our results indicated that Z-VAD-FMK alone did not significantly change the cell viability and LDH release but CA induced the decrease of cell viability and increase of LDH cytotoxicity in NCI-N87 and MKN-74 cells; however, Z-VAD-FMK inhibits the effects of CA on cell viability and LDH cytotoxicity GC cells (Fig. 1f, g). Furthermore, to further identify relationship between CA and apoptosis, we performed Western blotting analyses. Z-VAD-FMK treatment inhibits caspase-3 cleavage and CA alone mediates caspase-3 cleavage in CA-induced GC cells; however, Z-VAD-FMK suppresses caspase-3 cleavage in CA-treated GC cells (Fig. 1h). These results indicated that CA causes apoptotic cell death in GC cells.

### CA mediates autophagy in GC cells

**Fig. 2:**
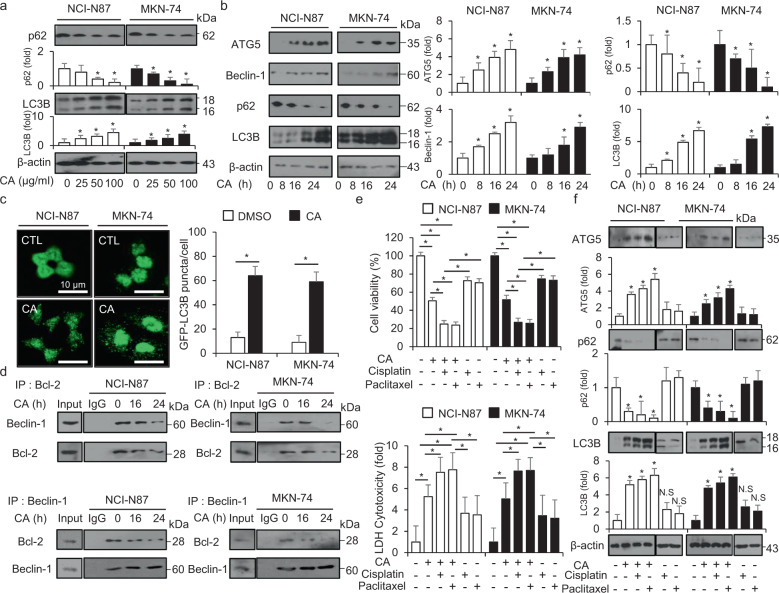
Autophagy activation in cinnamaldehyde-treated GC cells. **a**, **b** Western blot analysis of ATG5, Beclin-1, p62, and LC3B protein levels in NCI-N87 and MKN-74 cells treated with CA for the indicated doses and times. **c** NCI-N87 and MKN-74 cells transfected using the pEGFP-LC3 vector were treated with CA (50 μg/mL) for 24 h. Fluorescence microscopy analyses calculated the numbers of fluorescent puncta; *P < 0.05. **d** NCI-N87 and MKN-74 cells were treated with CA (50 μg/mL) for the indicated times (0, 16, and 24 h). Bcl-2 was immunoprecipitated in NCI-N87 and MKN-74 cells, and these proteins were subjected to Western blotting analyses. Beclin-1 was detected in immunoprecipitates prepared with anti-Bcl-2 antibody by immunoprecipitation. Bcl-2 was also identified in immunoprecipitates prepared with anti-Beclin-1 antibody by immumoprecipitation. **e** After NCI-N87 and MKN-74 cells were treated with cisplatin (5 μM) or paclitaxel (50 nM) in combination with CA (50 μg/mL) for 24 h, WST-1 and LDH assays were performed; *P < 0.05. **f** Protein samples were loaded for Western blotting analyses for p62, ATG5, and LC3B. β-actin was used as a protein loading control.

To identify whether CA regulates autophagy in GC cell, we performed Western blotting analysis which would determine the expression of the autophagy-related genes, LC3B and p62. It was found that CA causes dose-dependent increase of LC3-II and decrease of p62 in NCI-N87 and MKN-74 cells (Fig. 2a). To further analyze the autophagy effects of CA in NCI-N87 and MKN-74 cells, Western blotting analysis was performed. These results showed that CA treatment powerfully downregulated the expression of p62 and upregulated the expression of ATG5, Beclin-1 and LC3-II (Fig. 2b). To confirm autophagic vacuoles by CA treatment, pEGFP–LC3 vector was transiently transfected into both NCI-N87 and MKN-74 cells. Control cells have low LC3 puncta, whereas NCI-N87 and MKN-74 cells by CA treatment have high LC3 puncta (Fig. 2c). Anti-apoptotic Bcl-2 interacts with the autophagy-related protein Beclin-1 in ER lumen, and both Bcl-2 and Beclin-1 dissociate with the initiation of the autophagy process [41]. To investigate whether Bcl-2 interacts or dissociates with Beclin-1 in CA-treated GC cells, we performed co-immunoprecipitation (Co-IP) assay in CA-treated GC cells. When we performed the Co-IP assay with Bcl-2 antibody, Bcl-2 bound with Beclin-1 in GC cells, whereas CA treatment suppressed the interaction between Bcl-2 and Beclin-1. Furthermore, in the Co-IP assay with Beclin-1 antibody using lysates from NCI-N87 and MKN-74 cells, Beclin-1–Bcl-2 interaction was also disturbed by CA treatment (Fig. 2d). Therefore, CA treatment in GC cells leads to autophagy activation via the interruption of the Bcl-2/Beclin-1 complex. To identify synergistic autophagy effects of the combination of CA with cisplatin (5 µM) or paclitaxel (50 nM) in GC cells, we evaluated cell viability, LDH release, and the expression of autophagy-related genes such as p62, ATG5, and LC3B. Consequently, compared to CA alone, both CA + cisplatin and CA + paclitaxel decreased cell viability, enhanced LDH release, and induced the autophagy process, such as the upregulation of ATG5 and LC3B and the decrease of p62 expression (Fig. 2e and f). Taken together, our findings indicate that CA + cisplatin or CA + paclitaxel treatments induce synergic autophagic cell death in GC cells.

### Cinnamaldehyde modulates the autophagic flux in GC cells

**Fig. 3:**
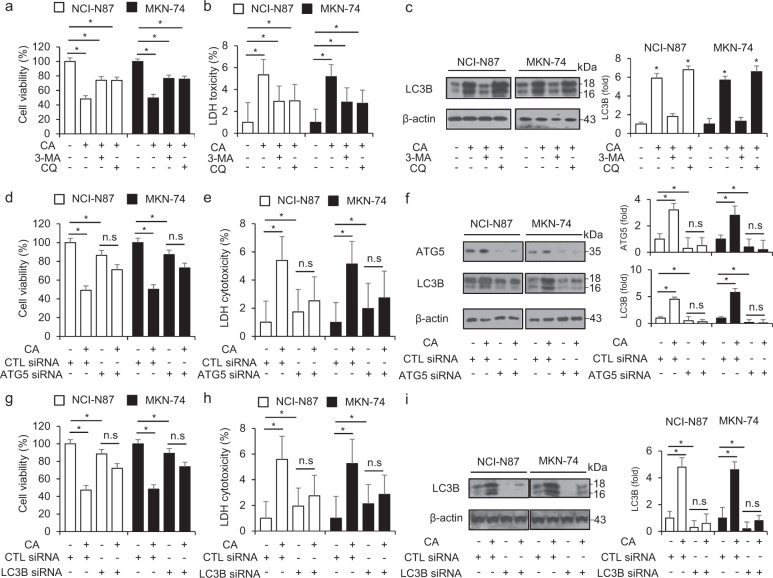
Targeting autophagy in cinnamaldehyde-treated GC cells. **a**, **b** Cell viability and LDH release were analyzed using WST-1 and LDH assays in CA (50 μg/mL, 24 h)-treated NCI-N87 and MKN-74 cells with 3-MA (5 mM) or CQ (20 μM) treatment for 24 h; *P < 0.05. **c** Western blot analysis of LC3B in the CA (50 μg/mL, 24 h)-treated NCI-N87 and MKN-74 cells with 3-MA (5 mM) or CQ (20 μM) for 24 h. **d**–**i** After NCI-N87 and MKN-74 cells were transfected with ATG5 and LC3B siRNAs, cell viability analysis, the LDH assay, and Western blotting analyses for ATG5 and LC3B were performed in CA (50 μg/mL, 24 h)-treated NCI-N87 and MKN-74 cells. β-actin was used as a protein loading control.

We investigated whether CA-induced autophagy is regulated by autophagy inhibitors such as 3-MA (early-stage autophagy inhibitor, 5 mM) and CQ (late-stage autophagy inhibitor, 20 µM) in GC cells. Cell viability analysis and Western blot analyses were analyzed in CA (50 μg/mL, 24 h), 3-MA (5 mM, 24 h) or CQ (20 μM, 24 h)-treated NCI-N87 and MKN-74 cells, respectively. In cell viability analysis, CA induced the reduction of cell viability, 3-MA and CQ did not change it (Supplementary Fig. 1a). In Western blot analyses, both CA and CQ treatment increase LC3II expression levels, but 3-MA alone decreases the expression levels of LC3I and LC3II (Supplementary Fig. 1b). Furthermore, we treated with CA in 3-MA or CQ-treated NCI-N87 and MKN-74 cells and performed cell viability analyses, the LDH assay, and Western blotting analysis. It was found that both 3-MA and CQ induce the restoration of cell viability and the inhibition of LDH release in CA-treated GC cells (Fig. 3a, b). In Western blotting analysis, 3-MA inhibited the expression of LC3-II indicating the disturbance of autophagosome formation, whereas both CA and CQ mediated the accumulation of LC3-II, indicating the disturbance of autophagosome formation and the interruption of the fusion of autophagosomes with lysosomes (Fig. 3c). 3-MA decreased LC3B expression in CA-treated NCI-N87 and MKN-74 cells, whereas CQ exerted the accumulation of LC3-II (Fig. 3c). Our result suggested that CA modulates the autophagic flux in GC.

### Autophagy knockdown inhibits CA-induced cell death in GC cells

To confirm the knockdown of autophagy-related genes, NCI-N87 and MKN-74 cells were transfected with ATG5- and LC3B-specific siRNA, respectively, and cultured with CA treatment for 24 h; then they were subjected to cell viability analyses, the LDH assay, and Western blotting analysis. The effects of CA on cell viability and LDH release were reversed in ATG5 and LC3B knockdown NCI-N87 and MKN-74 cells compared to CTL knockdown cells (Fig. 3d, e, g, h). Furthermore, the upregulation effects on ATG5 and LC3B expression by CA were inhibited in ATG5 and LC3B knockdown NCI-N87 and MKN-74 cells (Fig. 3f, i). These results indicate that targeting autophagy disturbs autophagic cell death in CA-treated NCI-N87 and MKN-74 cells.

### mTOR-AMPKα-ULK1 modulates cinnamaldehyde-mediated autophagic cell death in GC cells

**Fig. 4:**
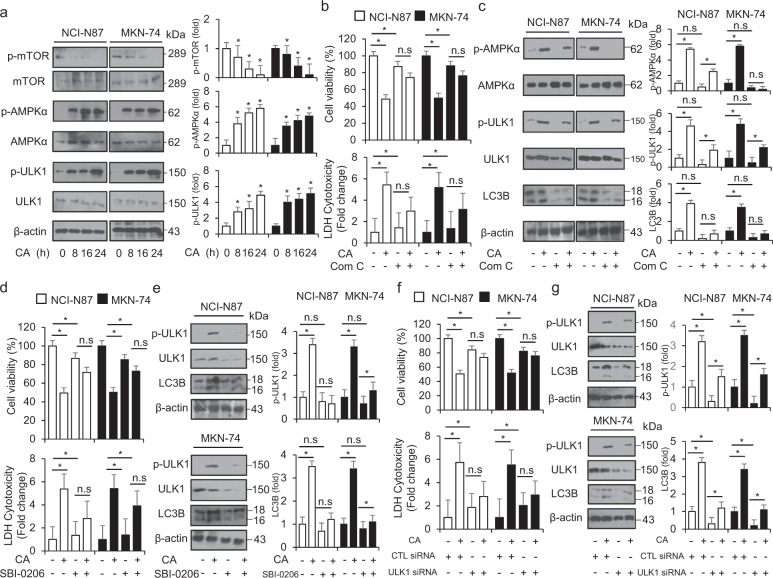
Inhibition of AMPKα/ULK1 promotes cell survival in cinnamaldehyde-treated GC cells. **a** NCI-N87 and MKN-74 cells were treated with CA (50 μg/mL) in a time-dependent manner (0, 8, 16, and 24 h). Cell lysates were loaded for Western blotting assay and analyzed with autophagy pathway-related antibodies including p-mTOR (Ser2448), mTOR, p-AMPKα (Thr172), AMPKα, p-ULK1 (Ser555), and ULK1. β-actin was used as a protein loading control. **b**, **c** The cell viability, LDH release, and protein expression of p-AMPKα, AMPKα, p-ULK1, ULK1, and LC3B in CA (50 μg/mL, 24 h)-treated NCI-N87 and MKN-74 cells in the presence or absence of compound C (2 μM, 24 h); *P < 0.05. **d**, **e** The cell viability, LDH release, and protein expression of p-ULK1, ULK1, and LC3B in CA (50 μg/mL, 24 h)-treated NCI-N87 and MKN-74 cells in the presence or absence of SBI-0206965 (SBI-0206 10 μM, 24 h); *P < 0.05. **f**, **g** Cell viability, LDH release, and protein expression of p-ULK1, ULK1, and LC3B in NCI-N87 and MKN-74 cells treated with CA (50 μg/mL, 24 h) in the presence or absence of ULK1 siRNA (30 nM, 24 h); *P < 0.05.

To investigate the hypotheses regarding CA-induced autophagic cell death, the mTOR–AMPK–ULK1 axis in CA-treated NCI-N87 and MKN-74 cells was identified using Western blotting analysis. We demonstrated that the expression of p-AMPKα and p-ULK1 was significantly enhanced in a time-dependent manner, whereas the expression p-mTOR was downregulated by CA treatment at the indicated times (Fig. 4a). We questioned if compound C regulates CA-induced autophagic cell death by interrupting AMPKα. We co-treated NCI-N87 and MKN-74 cells with compound C and CA and performed cell viability, LDH assay and Western blotting analysis. It was found that compound C inhibited the reduction of cell viability and LDH release in CA-induced NCI-N87 and MKN-74 cells (Fig. 4b). Compound C inhibited the expression of p-AMPK, ULK1, and LC3B in CA-treated NCI-N87 and MKN-74 cells (Fig. 4c). Based on these findings, it was hypothesized that targeting ULK1 using a pharmacological inhibitor and specific siRNA may regulate CA-induced autophagic cell death in GC cells. SBI-0206965, a pharmacological ULK1 kinase inhibitor, disturbs the phosphorylation of ULK1 [42]. To investigate whether the small molecule SBI-0206965 would affect autophagic cell death in CA-treated GC cells, we treated NCI-N87 and MKN-74 cells with SBI-0206965 and CA and performed cell viability analyses, the LDH assay, and Western blotting analysis. Consequently, SBI-0206965 was found to inhibit the decrease of cell viability and the production of LDH cytotoxicity in CA-mediated NCI-N87 and MKN-74 cells (Fig. 4d). Moreover, SBI-0206965 was found to interrupt LC3B expression by suppressing the phosphorylation of ULK1 in CA-treated GC cells (Fig. 4e). After ULK1 was knocked down by specific-siRNA in NCI-N87 and MKN-74 cells, GC cells were subjected to CA treatment. On performing cell viability analysis, the LDH assay, and Western blotting analysis, we found that ULK1 knockdown inhibited the reduction of cell viability and the increase of LDH cytotoxicity in CA-treated NCI-N87 and MKN-74 cells (Fig. 4f). In addition, in Western blotting analysis, ULK1 knockdown downregulated LC3-II expression by inhibiting ULK1 activity in CA-mediated NCI-N87 and MKN-74 cells (Fig. 4g). These results suggested that targeting AMPK and ULK1 regulates CA-induced autophagic cell death in GC cells.

### CA induces autophagic cell death via PERK–ATF4–CHOP signaling in GC cells

**Fig. 5:**
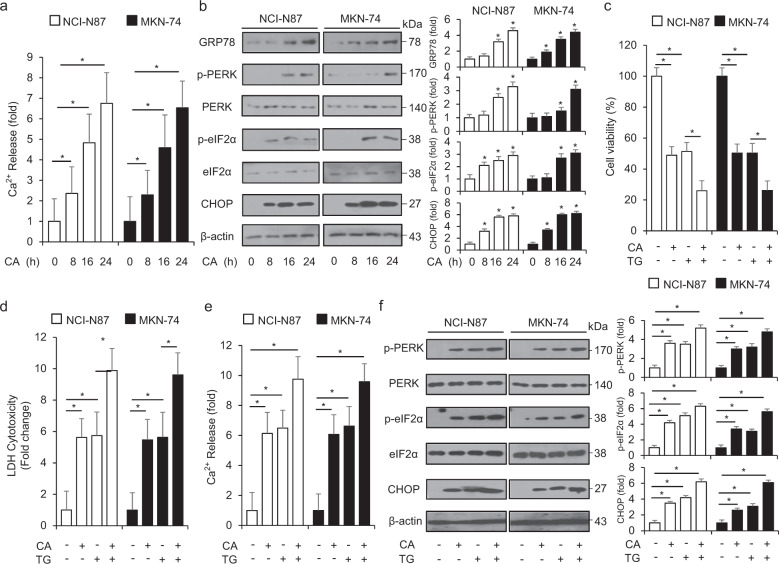
Cinnamaldehyde regulates cell death via ER stress response. **a** After NCI-N87 and MKN-74 cells were treated with CA (50 μg/mL) for the indicated times, intracellular calcium (Ca^2+^) assay was performed on the indicated times; *P < 0.05. **b** The activation of ER stress signaling, including GRP78, p-PERK, PERK, p-eIF2α, eIF2α, and CHOP, was investigated using Western blotting assay. **c**–**e** NCI-N87 and MKN-74 cells were treated with TG (3 mM; 24 h) and CA (50 μg/mL, 24 h), and cell viability, LDH release, and Ca^2+^ release were determined using WST-1, LDH, and intracellular calcium assays, respectively; *P < 0.05. **f** PERK signaling, including p-PERK, PERK, p-eIF2α, eIF2α, and CHOP, was investigated by Western blotting analyses. β-actin was used as a protein loading control.

Calcium (Ca^2+^) was restored in ER lumen and differential intracellular calcium release has a functional role in understanding biological molecular mechanisms involved in ER stress‐induced apoptosis and cell death, and the modification of Ca^2+^ ion concentration is a critical initiator of ER stress-related pathways such as PERK–ATF4 and IRE1α–JNK pathways [43]. To determine the changes in Ca^2+^ concentration in CA-treated GC cells, we performed the calcium assay in a time-dependent manner. The results indicated that CA treatment enhances fluorescent intensity meaning Ca^2+^ release in NCI-N87 and MKN-74 cells (Fig. 5a). These findings suggested that the increased Ca^2+^ release regulates CA-induced ER stress in GC cells. To further investigate whether ER stress is associated with CA-mediated cell death, we screened the PERK axis of the ER stress pathway in a time-dependent manner in CA-treated GC cells. CA induced the expression of GRP78, p-PERK, p-eIF2α, and CHOP in NCI-N87 and MKN-74 cells (Fig. 5b). To investigate the effect of CA on ER stress in GC cells, we performed cell viability analysis, the LDH assay, the calcium assay, and Western blotting analysis with ER stress inducer TG and CA treatment. When compared to CA or TG alone, TG in combination with CA induces further reduction of cell viability and the increase of LDH and Ca^2+^ release (Fig. 5c–e). In Western blotting analysis, both TG and CA treatments further increased the expression of p-PERK, p-eIF2α, and CHOP in NCI-N87 and MKN-74 cells compared to TG or CA alone (Fig. 5f). These results suggested that CA induces the PERK-CHOP axis-medicated cell death through calcium ion release in GC cells.

### PERK inhibition suppresses CA-induced autophagic cell death in GC cells

**Fig. 6:**
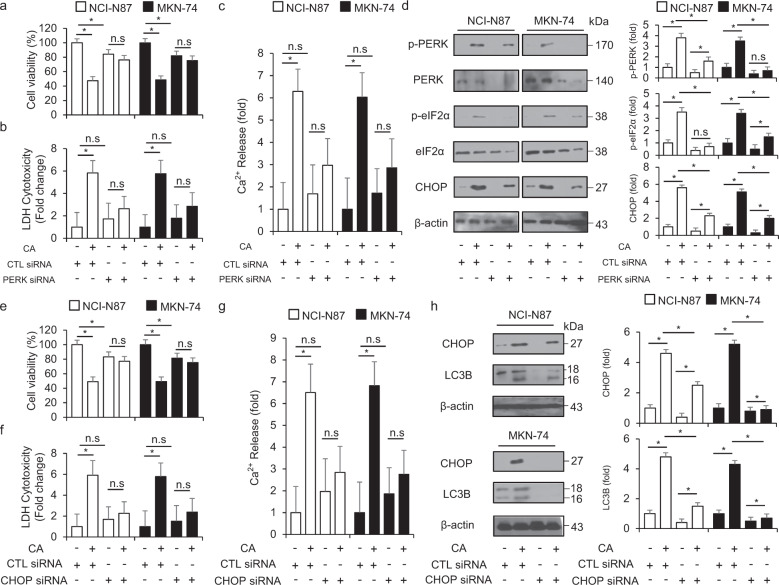
Targeting ER stress blocks cell death through the inhibition of autophagy in cinnamaldehyde-induced GC cells. **a**–**d** NCI-N87 and MKN-74 cells were transfected with PERK siRNA and treated with CA (50 μg/mL, 24 h). Cell viability, LDH release, and Ca^2+^ release were determined by WST-1, LDH, and Ca^2+^ assays, respectively; *P < 0.05. p-PERK, PERK, p-eIF2α, eIF2α, and CHOP were detected using Western blotting analyses. β-actin was used as a protein loading control. **e**–**h** NCI-N87 and MKN-74 cells were transfected with control or CHOP siRNA in the presence or absence of CA (50 μg/mL, 24 h). Cell viability, LDH release, and Ca^2+^ release were determined by WST-1, LDH and Ca^2+^ assays, respectively; *P < 0.05. The protein expression of CHOP and LC3B was detected using Western blotting analyses. β-actin was used as a protein loading control; *P < 0.05.

In normal conditions, PERK, a powerful ER stress sensor, exerts a suppressive effect via the interaction with GRP78; however, the PERK–eIF2α–ATF4–CHOP axis induces by suppressing the binding with GRP78 [44]. It was hypothesized that targeting PERK by specific siRNA may modulate CA-induced ER stress-mediated cell death in GC cells. After PERK and CHOP were transfected by specific-siRNA in NCI-N87 and MKN-74 cells, CA treatment was done. When we performed cell viability analysis, the LDH assay, the Calcium assay and Western blotting analysis, PERK and CHOP knockdown were found to suppress the decrease of cell viability and the increase of LDH cytotoxicity and calcium release in CA-treated NCI-N87 and MKN-74 cells (Figs. 6a–c, e–g). With Western blotting analysis, PERK and CHOP knockdown led to decreased p-PERK, p-eIF2α, and CHOP expression by blocking PERK in CA-treated NCI-N87 and MKN-74 cells (Fig. 6d, h). These results suggested that PERK and CHOP knockdown suppresses ER stress-mediated cell death in CA-induced GC cells.

### G9a inhibition regulates cinnamaldehyde-induced autophagic cell death in GC cells

**Fig. 7:**
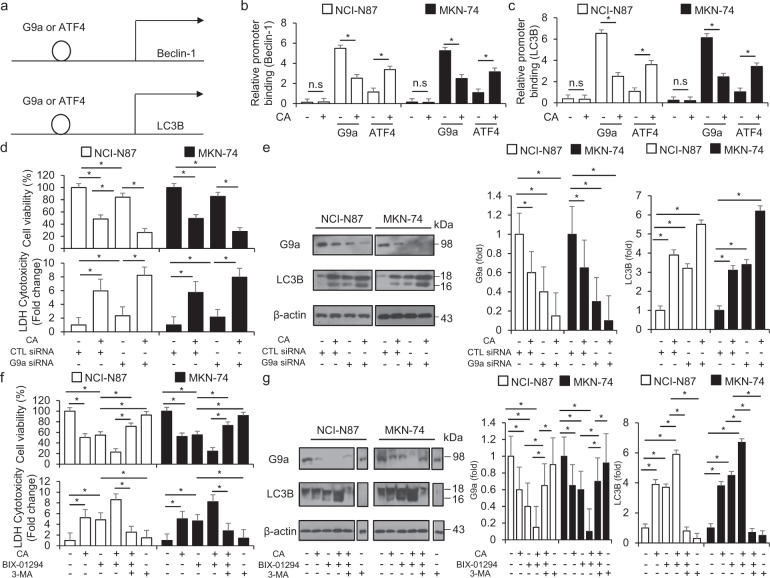
Targeting G9a modulates autophagic cell death in cinnamaldehyde-mediated GC cells. **a** The localization of G9a and ATF4 on the LC3B and Beclin-1 promoter. **b**, **c** CA regulates G9a and ATF4 binding on the Beclin-1 and LC3B promoter. CA (50 μg/mL, 24 h) treatment was performed in NCI-N87 and MKN-74 cells, and real-time ChIP assays of the LC3B and Beclin-1 promoter region were performed with G9a and ATF4 antibodies. **d**, **e** NCI-N87 and MKN-74 cells were transfected with control or G9a siRNA based on the presence or absence of CA. Cell viability and LDH release were determined by WST-1 and LDH assays, respectively; *P < 0.05. Protein expression of G9a and LC3B was detected using Western blot assay. β-actin was used as a protein loading control. **f**, **g** NCI-N87 and MKN-74 cells were treated with CA (50 μg/mL, 24 h) and/or BIX-01294 (10 μM, 24 h) and 3-MA (5 mM, 24 h). Cell viability was determined by WST-1 assay, and LDH release was determined by LDH assay. Protein levels of G9a and LC3B were detected using Western blotting. β-actin was used as a protein loading control; *P < 0.05

Recent reports indicate that histone modification modulates autophagy flux and is associated with early and late stages, including H3K9me2 and H4K16 deacetylation [45]. The active ingredient aqueous cinnamon extract and its active ingredients, such as cinnamaldehyde, cinnamyl alcohol, and cinnamic acid, regulate HDAC activity [46]. BIX-01294, a G9-specific inhibitor, mediates the reduction of H3K9me2 via G9a inhibition and induces autophagic cell death through the AMPK axis [47]. To identify whether CA treatment regulates the G9a expression in GC cells, we performed the immunofluorescence assay. Our results suggested that CA inhibits the G9a expression (Supplementary Fig. 2). To validate G9a and ATF4 binding on Beclin-1 and LC3B proximal promoter in CA-treated GC cells, we performed the ChIP assay from DNA fragments by sonication. In the real-time ChIP assay, the binding sites associated with Beclin-1 and LC3B genes were bound by G9a before CA treatment in NCI-N87 and MKN-74 cells, respectively, and CA treatment inhibited G9a binding and induced ATF4 binding on Beclin-1 and LC3B promoter (Fig. 7a–c). To determine if G9a is associated with CA-mediated autophagic cell death, after NCI-N87 and MKN-74 cells were knocked down by G9a-specific siRNA, CA treatment was performed. In addition, when we performed cell viability analysis, the LDH assay, and Western blotting analysis, cell viability was more decreased by CA in G9a knockdown cells than in control cells, and LDH release was more increased in CA-treated G9a knockdown cells than in control cells (Fig. 7d). Western blotting analysis revealed that G9a knockdown cells had upregulated LC3-II expression, and CA treatment also blocked G9a levels and increased LC3-II levels (Fig. 7e). To investigate whether co-treatment with CA and BIX-01294 is associated with autophagic cell death in CA-treated NCI-N87 and MKN-74 cells, we performed the cell viability assay, the LDH assay, and Western blotting analysis. Cell viability was significantly decreased by CA or BIX-01294 treatment, and LDH cytotoxicity was increased after CA or BIX-01294 treatment; however, CA + BIX-01294 showed further decreased cell viability and enhanced LDH release in NCI-N87 and MKN-74 cells (Fig. 7f). Furthermore, the synergic effects of CA+BIX-01294 on cell viability and LDH release were reversed in 3-MA-pretreated NCI-N87 and MKN-74 cells (Fig. 7f). Western blotting suggested that CA + BIX-01294 downregulated the G9a expression and upregulated the LC3B-II level in NCI-N87 and MKN-74 cells (Fig. 7g). In addition, 3-MA inhibited the reduction of G9a and the increase of LC3-II in CA- and/or BIX-01294-treated NCI-N87 and MKN-74 cells (Fig. 7g). In conclusion, these results indicate that CA + BIX-01294 treatment induces autophagic cell death by inhibiting G9a, whereas autophagy inhibition suppresses CA + BIX-01294-induced autophagic cell death through the restoration of G9a. Therefore, our findings suggest that G9a is essential for CA-induced autophagic cell death in GC cells.

## Discussion

Many researchers have suggested that flavonoids, including kaempferol, apigenin, and quercetin, can be used as potential anti-cancer drugs owing to their potent anti-tumor effect and lesser side effects [48–50]. Although flavonoids are one of many studied phytochemicals, detailed biological experiments have encouraged to diverse disease therapeutics. Previous reports against the role of cinnamaldehyde, a flavonoid isolated from *C. cassia*, can be a novel anti-cancer drug, but there is a lack of knowledge about detailed biological and molecular mechanisms of CA. Cinnamaldehyde, a well-known HDAC inhibitor and anti-tumor reagent, exerts powerful anti-cancer effect through apoptosis and cell death in various cancer types, including colorectal cancer, non-small cell lung cancer, head and neck cancer, hepatocarcinoma, and leukemia [51, 52]. Programmed cell death type II (Autophagic cell death) was reported to have differential mechanism and signaling programmed cell death type I (Apoptosis) [53]. Unlike apoptosis, autophagic cell death induces cell death by producing ROS, losing plasma membrane integrity, and membrane oxidation degradation and then mediates cell death via the activation of many caspases, including caspase−2, −3, −6, 8, 9, and 10 [54, 55]. Furthermore, Bcl family proteins such as Bcl-2 and Bcl-X_l_ play a potential role to determine differential mechanism between apoptosis and autophagic cell death [56]. Bcl-2 blocks apoptosis process by inhibiting the activation of Bax and the release of cytochrome *c* [57]. Bcl-2 on the endoplasmic reticulum (ER) suppresses autophagy process by interacting with beclin-1 and activates autophagy process by dissociating with beclin-1 [58]. Recent reports suggested that apoptosis machinery regulates autophagy process; however, autophagy-related proteins also modulate apoptosis process [59]. In this work, we show the first evidence that CA causes autophagy and cell death via the PERK–CHOP axis of ER stress pathways and the G9a binding on Beclin-1 and LC3B promoter in GC. *Cinnamomum cassia* extract (CCE) could suppress human oral cancer cell growth via caspase‐3 cleavage, Bcl‐2 reduction, and increasing autophagic markers, including LC3A, autophagy‐related protein 14, Rubicon and p62 in vivo and in vitro. However, autophagy inhibition increases apoptosis and cell death in CCE-treated human oral cancer cells, indicating that CCE induces protective autophagy and apoptosis [60]. Furthermore, 2’-hydroxycinnamaldehyde (HCA) also induces apoptosis and protective autophagy in human head and neck cancer cells [61]. However, we demonstrated that CA mediates autophagy and cell death by stimulating cleavage caspase-3; phosphorylating AMPKα, LC3B, Beclin-1, and ATG5; and downregulating p62 and mTOR phosphorylation in GC cells.

ER stress triggers diverse cell death-related pathways, such as intracellular Ca^2+^, MEKK1, ER membrane re-organization, and programmed cell death type II (autophagic cell death), in various cancer types [62–65]. Prolonged and excessive stimuli by flavonoids causes ER stress to initiate the apoptosis and autophagic cell death pathway [66]. PERK–eIF2*α*–ATF4–CHOP and IRE1–JNK/XBP1–CHOP signaling pathways are the two useful signposts of ER stress, which is regulated by ROS and calcium production [67]. Therefore, ER stress has been suggested as a potential strategy for diseases therapy. Recently, an emerging study has reported the use of phytochemicals, such as phenolic acids, stilbenes, tannins, coumarins, and flavonoids targeting ER stress, which have an anti-cancer effect [68]. Morusin, a flavonoid isolated in the root bark of *Morus australis* (Moraceae), has been suggested to activate ER stress-mediated cell death by enhancing GRP78, IRE1α, and CHOP as well as phosphorylation of eIF2α and Ca^2+^ ion production in epithelial ovarian cancer cells [69]. Baicalein causes ER stress-induced cell death in hepatocellular carcinoma cells. It upregulated the expression of IRE1α, CHOP and GRP78 and the phosphorylation of PERK, JNK and eIF2α through calcium release, suggesting that it mediates ER stress [70]. HCA, the selective hydrogenation of CA, upregulated the glucose-regulated protein 78 (GRP78), GADD153, heme oxygenase-1 (HMOX1), homocysteine-responsive ER-resident ubiquitin-like domain member 1 protein (HERPUD1), Bax, and cytochrome *c* in several human cancer cells, indicating that HCA induces ER stress-mediated cell death via ROS release [71]. We found that CA has shown significant anti-cancer activity and triggers cell death via the PERK–CHOP axis in GC cells. CA exerts the phosphorylation of PERK and eIF2*α* and the accumulation of ATF4 and CHOP in a dose- and time-dependent manner. Thapsigargin (TG), an ER stress inducer to raise the intracellular calcium levels, mediates higher cell death in CA-treated GC cells compared to cells treated with TG or CA alone. In addition, targeting ER stress, including PERK- and CHOP-specific siRNAs, inhibited CA-induced cell death. Herein, our results indicated that CA increased intracellular Ca^2+^ release and cell death through ER stress.

Hot topics in increasing reports indicate a biological mechanistic interplay between the ER stress and autophagy [72]. Both LC3B and p62 interact with each other and are central factors of the autophagosome for the autophagy process [73]. Recent reports suggested that inducing ATF4 in the ER stress pathway frequently induces autophagic cell death by binding with a cyclic AMP response element site on the proximal promoter of autophagy-related genes, including LC3B, ATG5, and p62 [74]. Euchromatic histone–lysine N-methyltransferase (G9a) has been studied to serve functional roles in cell differentiation and tumor growth, and G9a was found to be upregulated under hypoxia response [75]. Furthermore, G9a binds to the promoters of autophagy-related genes, such as LC3B, Beclin-1, HIF-1, and WIPI1, and its binding inhibits the autophagy process [76]. In the relationship among ER stress, autophagy, and G9a, we hypothesize that G9a may act as an activator by binding at Beclin-1 and LC3B promoter and that ATF4 binding may block G9a binding at LC3B promoter. HDAC inhibitor often exerts the downregulation of G9a via epigenetic modification, and both HDAC and G9a inhibitors mediate autophagic cell death [77]. BIX01294, a G9a inhibitor, induces autophagic cell death by dissociating G9a binding at LC3B promoter in colon cancer cells [78]. Our previous reports indicated that kaempferol, an inhibitor of HDAC and a flavonoid, mediates ER stress and autophagic cell death by inhibiting G9a binding at LC3 promoter in GC cells [79]. These findings suggested that CA induces ER stress and autophagic cell death by inhibiting G9a binding at LC3B promoter in GC cells.

## Conclusion

In conclusion, our results identified that CA mediates ER stress and autophagic cell death via the PERK–CHOP signaling pathway, inhibition of G9a binding on Beclin-1 and LC3B promoter, and dissociation of Bcl-2–Beclin-1 in GC cells. A broader study of the biological mechanism of CA may suggest useful anti-cancer therapeutic strategies.

## Supplementary information

Supplementary materials
