# Supplementary material for: Cinnamaldehyde induces autophagy-mediated cell death through ER stress and epigenetic modification in gastric cancer cells
Source: Acta Pharmacol Sin. 2021 May 12;43(3):712–23. doi: 10.1038/s41401-021-00672-x (PMC8888591; doi:10.1038/s41401-021-00672-x)

Supplementary materials:

Cinnamaldehyde induces autophagy-mediated cell death through ER stress and epigenetic modification in gastric cancer cells

Tae Woo Kim*

Department of Preventive Medicine, College of Korean Medicine, Kyung Hee University, Seoul, Korea.

* To whom correspondence should be addressed:

Tae Woo Kim*, Department of Preventive Medicine College of Korean Medicine, Kyung Hee University, 1 Hoegi, Seoul, 130-701. Korea. Tel: +82-2-961-0329; Fax: +82-2-961-1165; E-mail: tae1410@naver.com

Supplementary Figure 1. CA regulates autophagic flux in GC cells.

(A-B) Cell viability analysis and Western blot analyses were analyzed in CA (50 μg/mL, 24 h), 3-MA (5 mM, 24 h) or CQ (20 μM, 24 h)-treated NCI-N87 and MKN-74 cells, respectively; *, $p < 0.05$.

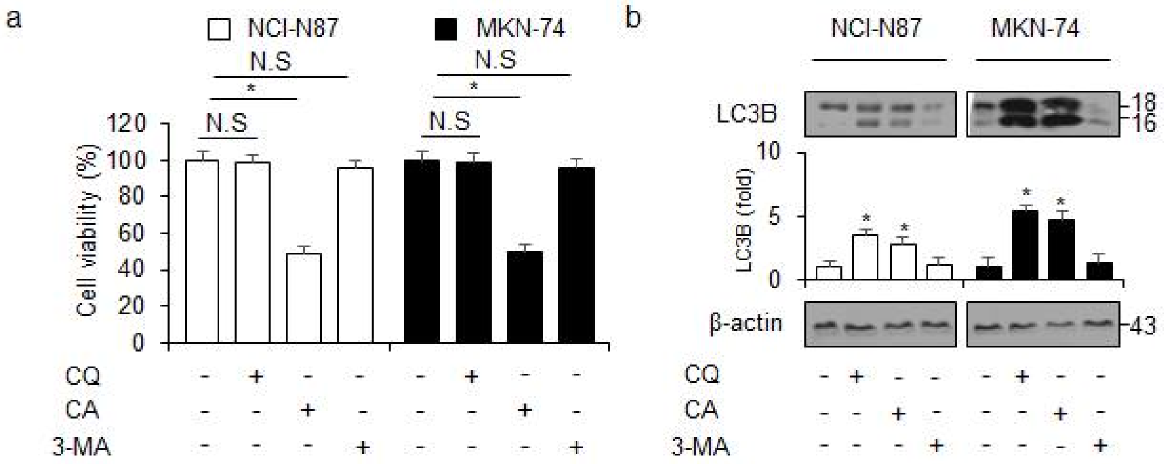

Supplementary Figure 2. CA treatment inhibits the G9a expression in GC cells.

(A) Immunofluorescence analysis indicated that CA inhibited G9a level; *, $p<0.05$. β-actin was used as the protein loading control.

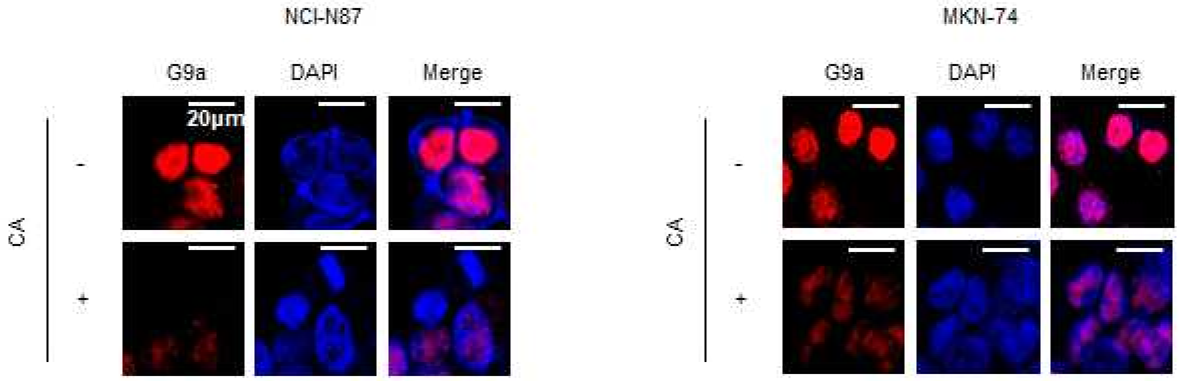

Supplement: Supplementary file 1 — Supplementary materials [file 41401_2021_672_MOESM1_ESM.pdf]
